# Responsive Inverse Opal Scaffolds with Biomimetic Enrichment Capability for Cell Culture

**DOI:** 10.34133/2019/9783793

**Published:** 2019-10-29

**Authors:** Changmin Shao, Yuxiao Liu, Junjie Chi, Jie Wang, Ze Zhao, Yuanjin Zhao

**Affiliations:** State Key Laboratory of Bioelectronics, School of Biological Science and Medical Engineering, Southeast University, Nanjing 210096, China

## Abstract

Three-dimensional (3D) porous scaffolds have a demonstrated value for tissue engineering and regenerative medicine. Inspired by the predation processes of marine predators in nature, we present new photocontrolled shrinkable inverse opal graphene oxide (GO) hydrogel scaffolds for cell enrichment and 3D culture. The scaffolds with adjustable pore sizes and morphologies were created using a GO and N-isopropylacrylamide dispersed solution as a continuous phase of microfluidic emulsions for polymerizing and replicating. Because of the interconnected porous structures and the remotely controllable volume responsiveness of the scaffolds, the suspended cells could be enriched into the inner spaces of the scaffolds through predator-like swallowing and discharging processes. Hepatocyte cells concentrated in the scaffold pores could form denser 3D spheroids more quickly via the controlled compression force caused by the shrinking of the dynamic scaffolds. More importantly, with a program of scaffold enrichment with different cells, an unprecedented 3D multilayer coculture system of endothelial-cell-encapsulated hepatocytes and fibroblasts could be generated for applications such as liver-on-a-chip and bioartificial liver. It was demonstrated that the resultant multicellular system offered significant improvements in hepatic functions, such as albumin secretion, urea synthesis, and cytochrome P450 expression. These features of our scaffolds make them highly promising for the biomimetic construction of various physiological and pathophysiological 3D tissue models, which could be used for understanding tissue level biology and *in vitro* drug testing applications.

## 1. Introduction

A three-dimensional (3D) cell culture confers a high degree of clinical and biological relevance to *in vitro* models [1–5]. Compared with conventional two-dimensional (2D) cell cultures, 3D cell cultures allow the cellular self-organization of appropriate extracellular matrix (ECM) assembly with complex cell-matrix and cell-cell interactions that mimic the functional properties of the corresponding tissue *in vivo* [6–11]. To realize the desired 3D cellular processes, numerous 3D biomimetic scaffolds that incorporate different biochemical, mechanical, or architectural cues have been developed for cell cultures [12–15]. Benefiting from their 3D structural features, these scaffolds have found various applications, such as cell therapy, basic organ physiology, drug discovery, and tissue engineering [16–18]. However, because of their polydispersity and the inability to control the degree of connectivity between their pores, most of these scaffolds could only provide limited external surfaces for random cell enrichment and attachment [19, 20]. In addition, as the component materials are nonresponsive, the resultant scaffolds are usually static and only provide stiff structures for cell cultures. Such structures bear little resemblance to biomimetic tissue microenvironments. Thus, the development of new scaffolds with controllable microstructures and dynamic stimuli-responsive features is still required to enable biomimetic 3D cell culture.

**Figure 1:**
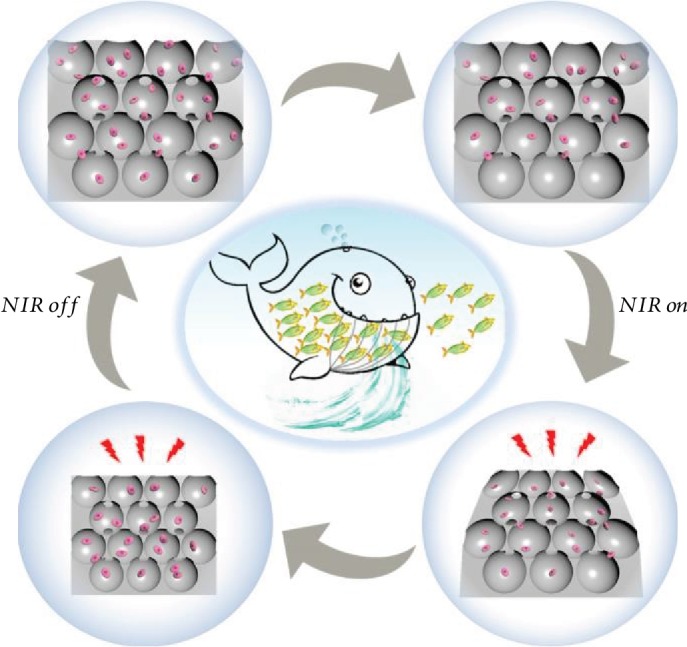
Schemes of the photocontrollable GO hydrogel scaffolds with cell enrichment capability.

Inspired by the predation processes of existing natural marine predators, in this paper we present new photocontrollable inverse opal graphene oxide (GO) hydrogel scaffolds with the desired features for cell capture and culture, as schemed in Figure 1. There are many predators in the ocean, ranging from small jellyfish to large whales. They swallow plankton and fish, along with large volumes of water, after which the water is discharged by controlled shrinking of the predators' bodies, and the nutrients remain. By repeating this process, large amounts of foods are accumulated to provide sufficient energy for their life. Thus, it is conceived to construct an intelligently shrinkable scaffold for cell enrichment by using stimuli-responsive materials as the elements. For this purpose, GO materials are good candidates due to their extraordinary physical and chemical properties, such as large specific surface area and abundant functional groups on the surface [21–23]. Thus, GO composited materials have been applied in a wide range of fields, including optoelectronic devices, flexible sensors, and life sciences [24, 25]. In particular, with the integration of biocompatible hydrogels, the GO composited materials could be imparted with distinct features of high hydrophilicity, large specific surface area, and physical analogue of extracellular matrix, all of which could facilitate their applications in cell culture and other biomedical applications [26, 27]. However, most of the GO composited materials are with uncontrollable pores, and their potential value for constructing cell-enrichable scaffolds is still unrealized.

Herein, we employed a microfluidic emulsion self-assembly approach for the generation of inverse opal GO hydrogel scaffolds for cell enrichment and 3D culture. Microfluidic techniques have emerged as advanced methods for fabricating microstructured materials due to their ability in executing precise operations on small quantities of fluids [28–34]. Thus, scaffolds with tunable pore sizes and morphologies could be generated by polymerizing and replicating the assembled microfluidic emulsions in the GO and N-isopropylacrylamide (NIPAM) dispersed solution. Due to the near infrared (NIR) absorption of GO and the thermally responsive shape transition of the NIPAM, the resultant scaffolds displayed a photothermally responsive shrinkage ability, which could enrich suspended cells through predator-like swallowing and discharging processes. It was demonstrated that hepatocyte cells could form denser 3D spheroids more quickly in the shrinkable scaffolds than those in unshrinkable methods, and an unprecedented 3D multilayer coculture system of endothelial-cell-encapsulated hepatocytes and fibroblasts could also be achieved by a program of scaffold enrichment with different cells. This multicellular system exhibited a significant improvement in hepatic functions as a liver-on-a-chip or bioartificial liver compared to the hepatocytes cultured alone.

## 2. Results and Discussion

**Figure 2:**
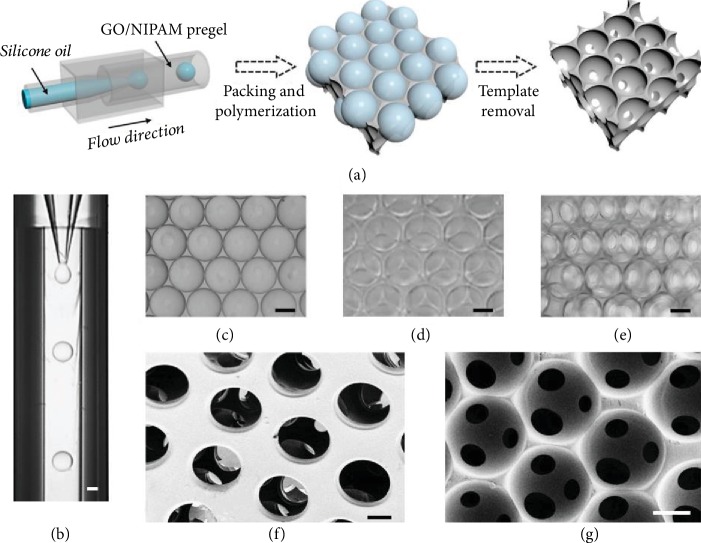
The structure of the GO hydrogel scaffolds. (a) Schematic illustration of the fabrication of the GO hydrogel scaffolds. (b) The generation of single-emulsion droplet templates. (c, d) The droplet templates orderly self-assemble into monolayer (c) and multilayer (d) packings. (e) Optical image of scaffolds. (f, g) SEM images of dehydrated scaffolds (top view and section view). The scale bar is 100 *μ*m.

In a typical experiment, the stimuli-responsive GO hydrogel bioscaffolds were generated by negatively replicating the emulsion droplet templates, as shown in Figure 2(a). A 3D glass capillary microfluidic device was employed for the preparation of the oil-in-water droplet templates, which were self-assembled into a hexagonal close-packed structure and formed an ordered droplet array under the influence of their density. The generated droplets possessed uniform size, good sphericity, and high monodispersity, which provided a reliable template for the inverse opal scaffolds (Figures [Supplementary-material supplementary-material-1] and [Supplementary-material supplementary-material-1]). This ordered packing of the emulsion droplets endowed the connectivity among all spherical cavities and a maximum theoretical porosity of 74%. After the formation of the ordered oil droplet template lattices, the outer phase of the GO/NIPAM pregel solution was polymerized into solid hydrogel scaffolds by ultraviolet (UV) irradiation. Then, the inverse opal-structured porous GO hydrogel scaffolds were obtained by removing the oil droplet templates. The resultant scaffolds were investigated as described in Figure 2. It could be observed that the scaffolds had an ordered and uniform porous structure under optical microscopy (Figures 2(b)–2(e)). In addition, in order to further verify their 3D structure and pore connectivity, the scaffolds were characterized by scanning electron microscopy (SEM), as shown in Figures 2(f) and 2(g). It was found that the scaffolds had a multilayer interconnected porous structure, which ensured the formation of open channels throughout the entire scaffolds and facilitated the nutrient transport. Moreover, the generated GO hydrogel scaffold had stable structural characteristics such as uniform pore size, good connectivity, and high porosity, which would lay a good foundation for its biological applications.

To make the GO hydrogel scaffolds suitable for cell culture, we have investigated and optimized their relevant physicochemical factors. Because the droplet templates were generated by microfluidics, the droplet sizes and their corresponding pore sizes in the scaffolds could be well controlled by adjusting the liquid flow rates. As shown in [Supplementary-material supplementary-material-1] and [Supplementary-material supplementary-material-1], it was found that the diameter of the droplets increased with the increasing flow rate of the inner phase, while the diameter decreased when the flow rate of the outer phase increased. Therefore, by adjusting the flow rates of the two phases, we could prepare scaffolds with different pore sizes. Afterward, the responsive ability of the GO/NIPAM composite hydrogel materials and the concentrations of the two components were also investigated ([Supplementary-material supplementary-material-1]). The results showed that the shrinkage property of the composite hydrogel was inhibited when the concentration of GO was too high or too low, as shown in [Supplementary-material supplementary-material-1]. Thus, the concentration of the GO component at 2.0 mg/mL was chosen in the following studies so as to impart the composite hydrogel with the best shrinkage property. Besides, when the concentration of GO was fixed, the shrinkage degree of the composite hydrogel clearly reduced with the increasing NIPAM concentration ([Supplementary-material supplementary-material-1]). Hence, considering the shrinkage performance and the mechanical strength of the composite hydrogel material, the optimized concentration of NIPAM at 15 wt% was chosen in subsequent studies. In addition, to make the GO/NIPAM hydrogel scaffolds more biocompatible, we also adjusted the lower critical solution temperature (LCST) of NIPAM by doping them with N-methylol acrylamide (NMAM). It was found that LCST increased with the increasing amount of NMAM, whereas the contractility decreased simultaneously ([Supplementary-material supplementary-material-1]). Finally, the NIPAM/NMAM ratio of 10 was chosen, and the corresponding LCST was around 40°C. Under these optimized conditions, the contractility of the GO hydrogel scaffolds was above 50% under NIR irradiation, and the specific dynamic shrinking process was shown in [Supplementary-material supplementary-material-1].

**Figure 3:**
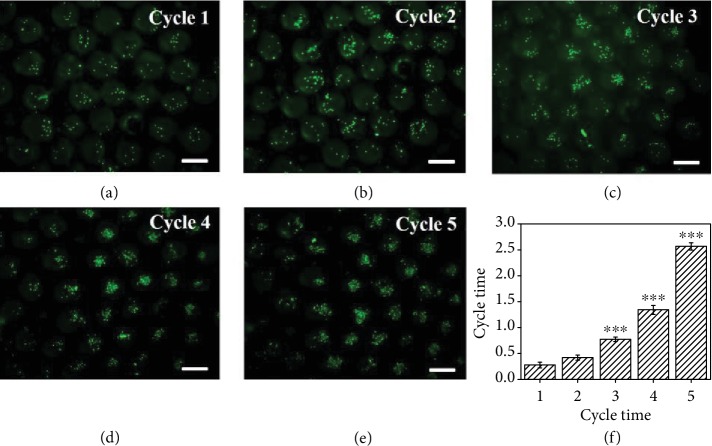
Cell enrichment process. (a–e) The fluorescent images of enriched cells in the scaffolds after 5 cycles. (f) Results of the MTT assay of the enriched cells after 5 cycles; ^∗∗∗^*p* < 0.01. The scale bar is 200 *μ*m.

To mimic the predation mechanism of marine predators and implement the construction of dynamic stimuli-responsive materials, we used the GO/NIPAM composite hydrogel as the skeleton material in the generation of the inverse opal-structured GO hydrogel scaffolds, which displayed a photothermally responsive shrinking ability because of the NIR absorption of GO and the shape transition triggered by the thermal response of the NIPAM hydrogel. Due to the interconnected porous structures and the remotely controllable volume responsiveness of the inverse opal-structured GO hydrogel scaffolds, suspended human hepatocarcinoma (HepG2) cells could be enriched into their inner spaces through the predator-like swallowing and discharging processes, as shown in the schematic in Figure 1. To verify this capacity of the GO hydrogel scaffolds, the scaffolds were first placed in a culture medium containing suspended HepG2 cells and then contracted from top to bottom under NIR radiation. During this process, the shrinkage of the upper pores of the scaffolds was just like closing the door, so that the cells were locked in the pores of the scaffolds. The culture medium in the scaffolds could be squeezed out through the shrinking process from the top to the bottom at the same time, and thus when the NIR was turned off and the door opened again, more cells would be sucked into the pores of the scaffolds. By repeating the above process, large amounts of cells would be enriched in the scaffolds (Figure 3), which contributed to the subsequent cell aggregate formation. Because of the good biocompatibility of the GO/NIPAM composite hydrogel, the resultant scaffolds were suitable for the growth and proliferation of different kinds of enriched cells, such as HepG2 cells ([Supplementary-material supplementary-material-1]).

It was worth mentioning that the sizes of the pores and the interconnected porous structure also decreased with the contraction of the scaffolds under NIR radiation. As shown in [Supplementary-material supplementary-material-1], the diameter of the pores was reduced from 200.13 ± 4.24 *μ*m to 105.38 ± 5.55 *μ*m and the diameter of the interconnected porous structure was reduced from 54.39 ± 2.03 *μ*m to 26.88 ± 0.89 *μ*m. Since a single suspended cell had a diameter of 10-15 *μ*m, it was easy for the suspended cells to enter the pores through the interconnected porous structure. Although the size of the interconnected porous structure after shrinkage was still larger than the suspended cells, it was more difficult for the cells to pass through the porous structure because they would easily accumulate during the contraction process. Thus, though there was a certain loss of cells during the contraction process, the outflow of cells was obviously smaller than the inflow of cells, which made cell enrichment easy to achieve. In addition, the uniformity of cell enrichment during the contraction process was also analyzed (Figures [Supplementary-material supplementary-material-1]–[Supplementary-material supplementary-material-1]). The results showed that the distribution of cells in the pores became more uniform with the increase of the number of contractions, which laid a good foundation for the subsequent formation of 3D cell spheroids.

**Figure 4:**
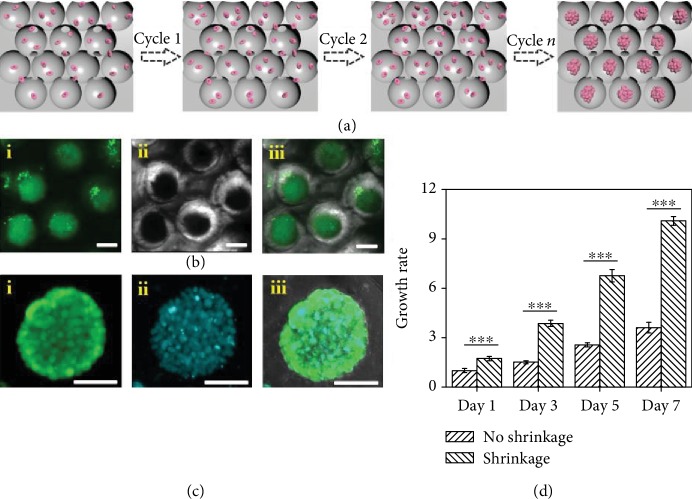
The HepG2 spheroids. (a) The process of formation of HepG2 spheroids. (b) The following confocal images are the HepG2 spheroids stained with calcein AM (green) after 7 days of culture: (i) the fluorescent view, (ii) the bright field view, and (iii) the merged view. (c) The following confocal images are the HepG2 spheroids stained with calcein AM (green) (i) and DAPI (blue) (ii) after 7 days of culture and (iii) their merged view. (d) Cell growth rate of the spheroids by the shrinkage and no shrinkage methods; ^∗∗∗^*p* < 0.01. The scale bar is 100 *μ*m.

To further exploit the functions of the GO hydrogel scaffolds, they were used for culturing 3D cell spheroids and we expected that the enriched cells would promote a more rapid formation of the denser 3D spheroids with a controlled compression force caused by the dynamic scaffold shrinkage (Figure 4(a)). To demonstrate this feature, the HepG2 cells were enriched into the scaffolds and observed by an optical microscope and a laser scanning confocal microscope (CLSM) through staining with calcein AM and 4,6-diamidino-2-phenylindole (DAPI) (Figures 4(b) and 4(c)). The results showed that cell seeding was successfully implemented by inoculating a certain density of cells suspended on the top of the scaffolds, and the spherical pores with uniform sizes also promoted the equal distribution of cells in each pore. As demonstrated in [Supplementary-material supplementary-material-1], we found that with the periodical shrinking of the scaffolds, the cell aggregates gradually increased with increasing incubation times, and 3D spheroids ultimately formed over a period of seven days. In addition, the viability of the cells in the center of spheroids was also observed by CLSM at intervals of 5 *μ*m in different *Z*-planes ([Supplementary-material supplementary-material-1]). It was found that the cell spheroids grown in the pores of the scaffolds had high viability, and the cells in both the exterior and interior parts of the spheroids were alive with excellent homogeneity of fluorescence intensity at different depths and locations. Moreover, the cell morphology was also significantly different in the case of spheroid formation in the scaffolds compared with conventional 2D cell cultures. Unlike the spindle-shaped cells that have been grown in flat multiwell plates, the edges of the cells in the scaffolds were smoother and more stereoscopic and were much more similar to the morphology *in vivo* ([Supplementary-material supplementary-material-1]). It was worth mentioning that the spheroids generated in this study were much denser and their formation was faster compared with those in unshrinkable methods (Figure 4(d)). This was exactly because our dynamic shrinkable scaffolds could compress large numbers of cells and accelerate the initial cell aggregation by a controlled compression force.

**Figure 5:**
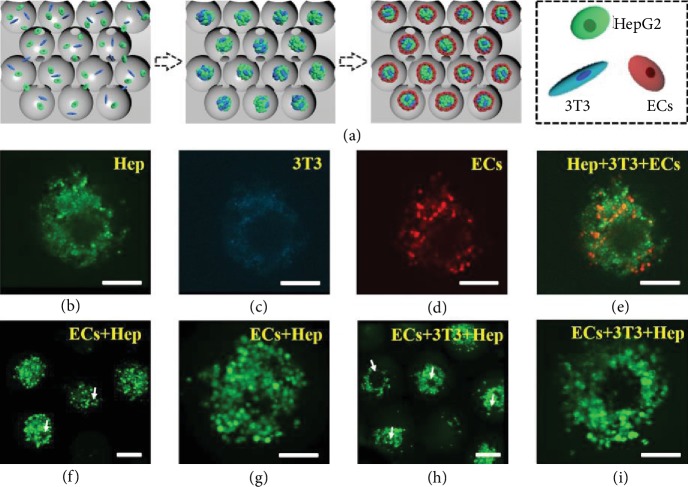
Cell coculture system. (a) Schematic diagram of the cell coculture system. (b–e) The confocal images of the cell coculture system containing HepG2 (green), 3T3 (blue), and ECs (red) on day 7 of culture. (f–i) The confocal images of calcein-AM-stained ECs after coculture with HepG2 and both 3T3 and HepG2 for 7 days. White arrows indicate capillary-like structures. The scale bar is 100 *μ*m.

More attractively, the aggregation of the spheroids and their interconnective channels are able to simulate the actual liver tissue where the lobules, i.e., the functional units of liver tissues, are connected by a network of blood vessels. For this purpose, by sequentially enriching different cells into the scaffolds, we demonstrated an unprecedented 3D multilayer coculture system of endothelial-cell-encapsulated hepatocytes and fibroblasts (Figure 5(a)). Specifically, three different kinds of cells, HepG2, mouse embryo fibroblast NIH3T3 (3T3), and human umbilical vein endothelial cells (ECs) were sucked into the porous scaffolds and cultured. Because of the stress caused by shrinkable scaffolds, these three kinds of cells were packed together to form a multilayer coculture system. To observe the distribution of cells in the scaffolds, these cells were stained with calcein AM (green), DAPI (blue), and DID cell-labeling solutions (red), respectively, before encapsulation. Figures 5(b)–5(e) demonstrates the CLSM images of cells after coculture in the scaffolds. It was found that cells seeded into the scaffolds showed good viability and indicated no apparent cell death for each type of cells during the seven-day coculture process.

It has been demonstrated that most methods for tissue engineering rely on the formation of new blood vessels from the host after implantation, but it is apparently insufficient for large and metabolic organs to meet the need for the endogenous growth of blood vessels. Therefore, it is necessary to induce the tubulous ECs in the reconstruction of liver structure *in vitro*, so as to provide sufficient blood vessels. In our program, the coculture system of hepatocytes with fibroblasts and ECs made it possible for ECs to form capillary-like structures. The CLSM images of calcein-AM-stained ECs after seven days of the coculture system are shown in Figures 5(f)–5(i) and [Supplementary-material supplementary-material-1], which indicated different tubule formation behaviors. The results showed that ECs cultured alone did not form tubule-like structures. Moreover, ECs indicated more tubular structures after coculture both with fibroblasts and hepatocytes than after coculture with either fibroblasts or hepatocytes alone, which indicated that ECs in the coculture scaffolds could be organized into tubule-like structures and could contribute to the reconstruction of liver tissue.

**Figure 6:**
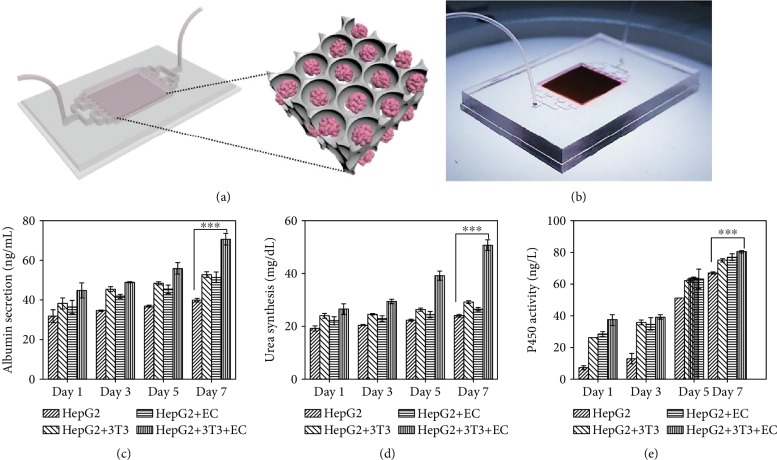
The applications of the GO hydrogel scaffolds in a liver-on-a-chip system. (a) Schematic of the construction of the liver-on-a-chip. (b) Image of the GO hydrogel scaffold-integrated liver-on-a-chip. (c–e) Albumin secretion (c), urea synthesis (d), and cytochrome P450 expression (e) of HepG2 after coculture with 3T3, ECs, and both 3T3 and ECs in the liver-on-a-chip system for 7 days; ^∗∗∗^*p* < 0.01.

Taken together, the above results have indicated that our scaffolds could be applied to produce stable liver coculture models with EC proliferation and tubular structure formation *in vitro*, which demonstrates its extremely important values and great potentials in liver-on-a-chip, bioartificial liver, etc. To implement this concept, we combined the scaffolds with microfluidics to construct a liver chip (Figure 6(a)). Organ-on-a-chip is a kind of bionic system that can simulate the main functions of human organs on a microfluidic chip. In addition to the characteristics of miniaturization, integration, and low consumption of microfluidic technology, organ-on-a-chip technology can precisely control multiple system parameters, such as the chemical concentration gradient, fluid shear force, the construction of cell graphic culture, and the interaction between tissue-to-tissue and organ-to-organ interfaces, so as to simulate the complex structure, microenvironment, and physiological functions of human organs. Because of these advantages, organ-on-a-chip technology has been expected to be a biomimetic, efficient, and energy-saving tool for physiological research and drug development.

Specifically, in our liver-on-a-chip system, microfluidic channels were placed on each side of the chip, which could provide culture medium or drug solution to the cocultured liver tissue in the scaffolds (Figure 6(b)). The microfluidic channels could realize a dynamic cell culture through micropump irrigation, which was conducive to the stable supply of nutrients to cells and the timely discharge of wastes. In addition, compared with static culture, the dynamic environment of cells is more similar to that in the body. The responsive GO hydrogel scaffold was placed in the recess in the middle of the chip. It was observed that the scaffolds integrated in the chip still had light-controllable shrinkage performance ([Supplementary-material supplementary-material-1]), which guaranteed the subsequent applications of the liver chips. To certify the effectiveness of the liver-on-a-chip system, liver chips cocultured with fibroblasts and ECs were constructed and the liver-specific functions were determined by albumin secretion, urea synthesis, and cytochrome P450 expression. As shown in Figures 6(c)–6(e), compared with the hepatocytes cultured alone, the coculture of hepatocytes with either fibroblasts or ECs and the coculture with both fibroblasts and ECs were all found to display increasing albumin secretion, urea synthesis, and cytochrome P450 expression. Among them, the three indicators in the group of coculture with both fibroblasts and ECs for seven days were significantly higher than those of hepatocytes cocultured with either of these cells or cultured alone (*p* < 0.01). These results indicated that the coculture of the three different kinds of cells in our liver-on-a-chip system could maintain liver-specific functions *in vitro*, which made the artificial bionic liver tissue possible. These results demonstrated the effectiveness of the proposed liver model for *in vitro* visualizable biological research, drug evaluation, and drug screening.

## 3. Conclusion

We have demonstrated a simple microfluidic emulsion self-assembled method for generating a novel kind of photocontrolled shrinkable inverse opal GO hydrogel scaffolds for cell enrichment and 3D culture. The resultant scaffolds displayed a photothermally responsive shrinking ability because of the NIR absorption of GO and the thermally responsive shape transition of the NIPAM hydrogel. Benefitting from these features, suspended cells could be enriched into the porous structures of the scaffolds through the predator-like swallowing and discharging processes. It was found that the enriched hepatocyte cells could form denser 3D spheroids more rapidly due to the compression force caused by dynamic scaffold shrinking. More importantly, with a sequential enrichment of different cells, an unprecedented 3D multilayer coculture system of hepatocytes with encapsulated fibroblasts and endothelial cells could be achieved. It was demonstrated that the resultant multicellular system had a significant improvement of the capabilities of tubule formation. Finally, we integrated the scaffolds with microfluidics to construct different liver-on-a-chip systems and demonstrated the hepatic secretion functions successfully. We believe that such functional inverse opal GO hydrogel scaffolds can be served as a valuable tool for understanding tissue level biology and *in vitro* drug testing applications.

## 4. Materials and Methods

### 4.1. Materials

N-Isopropylacrylamide (NIPAM, 97%), N,N′-methylenebisacrylamide (Bis), poly(ethylene glycol) diacrylate (PEGDA, average molecular weight of 700), 2-hydroxy-2-methylpropiophenone (HMPP), and octadecyltrichlorosilane (OTS) were all purchased from Sigma-Aldrich, USA. N-Methylol acrylamide (NMAM) was bought from Aladdin Industrial Corporation, Shanghai, China. Graphene oxide (GO) aqueous solution was purchased from Nanjing XFNANO Materials Tech Co., Ltd., Nanjing, China. Poly(ethylene glycol)-block-poly(propylene glycol)-block-poly(ethylene glycol) (F108) and sodium dodecyl sulfate (SDS) were purchased from Sigma-Aldrich, USA. HepG2 cells and NIH 3T3 cells were obtained from the Cell Bank of the Chinese Academy of Sciences, Shanghai, China. Human umbilical vein endothelial cells (HUVECs) were obtained from Tongpai Biological Technology Co., Ltd., Shanghai, China. Fetal bovine serum without mycoplasma, penicillin-streptomycin double antibiotics, DMEM medium, 0.25% trypsin-EDTA, and PBS solution (pH 7.4) were purchased from Gibco, USA. Calcein AM was purchased from Molecular Probes Co. DID cell-labeling solutions were purchased from Invitrogen Co. 4,6-Diamidino-2-phenylindole (DAPI) was bought from Sigma-Aldrich, USA. MTT (98%) was obtained from J&K Scientific Ltd., Shanghai. Dimethyl sulfoxide (DMSO) was purchased from Sigma-Aldrich, USA. Deionized water was obtained from a Millipore Milli-Q system. All other chemical reagents were the best grade.

### 4.2. Fabrication of the Inverse Opal GO Hydrogel Scaffolds

The inverse opal GO hydrogel scaffolds were generated by polymerizing and replicating the assembled microfluidic emulsion droplet templates. Briefly, a glass capillary device was constructed by assembling three capillaries (one inner, one outer, and one square glass). The inner capillary (outer/inner diameter: 1/0.8 mm) was prepared by a capillary puller (Sutter Instrument, P-97) to have a tapered tip with an inner orifice of 70-80 *μ*m and was treated with a hydrophobic reagent (OTS). The outer (collection) capillary has an outer diameter of 1 mm and an inner diameter of 0.8 mm. Then, the inner and outer capillaries were coaxially assembled in a square capillary with an inner diameter of 1.05 mm (AIT Glass, Rockaway, NJ, USA). Finally, an epoxy resin was used to seal the device where needed. To fabricate the single-emulsion templates, all fluids were pumped into the capillary microfluidic device by syringe pumps (Harvard PHD 2000 series). During the generation of the emulsion droplets, a typical set of flow rates for the inner and outer phases was 0.2 mL/h and 2 mL/h, respectively. The inner oil phase was methyl silicone oil, and the outer water phase was a GO/NIPAM composite pregel solution. The pregel solution was composed of NIPAM (15% *w*/*v*), Bis (mass ratio 1/29 to NIPAM), PEGDA (2% *v*/*v*), F108 (2% *w*/*v*), SDS (2% *w*/*v*), NMAM (mass ratio 1/10 to NIPAM), and HMPP (1% *v*/*v*). The aqueous GO (2.0 mg/mL) was mixed into the solution. These fluids flowed via the corresponding capillaries, and the inner oil phase of methyl silicone oil was sheered into droplets by the outer water phase of the GO/NIPAM mixture solution at the orifice of the inner capillary. The droplets were collected into a collection container. Then, the droplets self-assembled into ordered lattices and were exposed to ultraviolet (UV) irradiation. With this treatment, the GO/NIPAM pregel solution was polymerized into solid hydrogel scaffolds. Finally, the porous GO/NIPAM hydrogel scaffolds were obtained by washing away the methyl silicone using alcohol.

### 4.3. Cell Culture

The HepG2, NIH 3T3, and HUVEC cells were incubated in DMEM medium supplemented with 10% fetal bovine serum and 1% penicillin-streptomycin. All cells were incubated in the incubator (Heracell 150, Thermo Fisher Scientific, USA) with 5% CO_2_ at 37°C. The GO hydrogel scaffolds were sterilized under UV light for more than 4 h and transferred into a 48-well plate (Corning, USA). For the generation of HepG2 cell spheroids, 50 *μ*L of the cell suspension at a concentration of 4 × 10^5^ cells/mL was dropped on top of the scaffolds, and then 400 *μ*L of media was gently added. Immediately after cell seeding, the scaffolds were irradiated under 0.5 W cm^−2^ NIR at around 15 s per cycle for 5 cycles to enrich the cells, and each cycle was 20 s apart. The total culture volume was maintained at 450 *μ*L, and 250 *μ*L of the media was renewed every day. After forming the spheroids, the cells were stained by 5 *μ*M calcein AM and 0.5 *μ*g/mL DAPI for observation. For the investigation of the cell coculture system, HepG2 (4 × 10^5^ cells/mL), NIH 3T3 (3 × 10^5^ cells/mL), and HUVEC (3 × 10^5^ cells/mL) cells were seeded and enriched into the scaffolds as described above. These different kinds of cells were stained by 5 *μ*M calcein AM, 0.5 *μ*g/mL DAPI, and 5 *μ*M DID, respectively, before seeding. A half-volume of the culture media was renewed daily. The cell proliferation rate was tested by MTT assay. Briefly, the cells were placed in a 48-well plate and then culture medium with 10% MTT solution was added which was dissolved in the PBS buffer (5 mg/mL) and incubated at 37°C for 4 h. The solution was removed and 400 *μ*L of DMSO was added to dissolve the formazan crystals after incubation. The absorbance was detected by a microplate reader (Synergy HT, BioTek, USA). The albumin secretion was measured by a Rat Albumin ELISA Kit (Abcam, UK). The urea synthesis was measured by a Urea Assay Kit (BioChain, USA). The activity of CYP450 was detected by a Human Cytochrome P450 3A4 Elisa Kit (Nanjing Jiancheng Bioengineering Institute, Nanjing, China).

### 4.4. Characterization

The generation of droplets in the capillary was observed by a microscope (Nikon SMZ745T) equipped with a camera (S-PRIF1, AOS Technologies AG). The optical images of the scaffolds were taken by an optical microscope (Olympus BX51). The fluorescent images of enriched cells were obtained with a fluorescence microscope (Olympus SZX16). Fluorescent images of the cell spheroid and cell coculture system in the scaffolds were taken by a Laser Scanning Confocal Microscope (Olympus FV10i). The microstructures of the scaffolds were characterized by a scanning electron microscope (SEM, Hitachi, S-300N).

### 4.5. Statistical Analyses

All quantitative data were presented as means ± standard deviations. Unpaired Student's *t*-tests were performed to determine statistical significance, and *p* values < 0.01 or 0.05 were considered statistically significant. All data were analyzed with SPSS version 11.0 (SPSS, Inc., Chicago, IL, USA).

## Supplementary Materials

Supplementary 1 Figure S1: the real-time microscopic images of the generation of single-emulsion droplets and the relationship between the droplet diameter and the flow rate. Figure S2: the photothermally responsive behavior of the GO/NIPAM composite hydrogel. Figure S3: the fluorescent images and the MTT result of the HepG2 cells cultured on multiwell plates and the GO/NIPAM hydrogel membranes. Figure S4: the size of the pores and the interconnected porous structure before and after NIR radiation and the distribution of the cells in the pores after 1, 3, and 5 cycles. Figure S5: the images of the HepG2 spheroid formation in scaffolds on day 1, day 4, and day 7 of culture and the MTT result of the HepG2 spheroid cultured for 7 days, compared with the multiwell plate. Figure S6: the confocal images of the HepG2 spheroid in different *Z*-planes. Figure S7: SEM images of the cell spheroid. Figure S8: the confocal images of calcein-AM-stained ECs after coculture with 3T3 for 7 days. Figure S9: the construction of the liver-on-a-chip.

Supplementary 2 Movie S1: the inverse opal GO hydrogel scaffold under NIR light.
