# Erratum to “Responsive Inverse Opal Scaffolds with Biomimetic Enrichment Capability for Cell Culture”

**DOI:** 10.34133/2022/9850304

**Published:** 2022-02-04

**Authors:** Changmin Shao, Yuxiao Liu, Junjie Chi, Jie Wang, Ze Zhao, Yuanjin Zhao

**Affiliations:** State Key Laboratory of Bioelectronics, School of Biological Science and Medical Engineering, Southeast University, Nanjing 210096, China

In the article titled “Responsive Inverse Opal Scaffolds with Biomimetic Enrichment Capability for Cell Culture” [1], there was an error in Figure 6e. The scale on the *y*-axis was written as 0-100, whereas it should have been 0-1000. This error was introduced during the production process, and the publisher apologises for this error. The corrected figure is shown below:

## Figures and Tables

**Figure 1:**
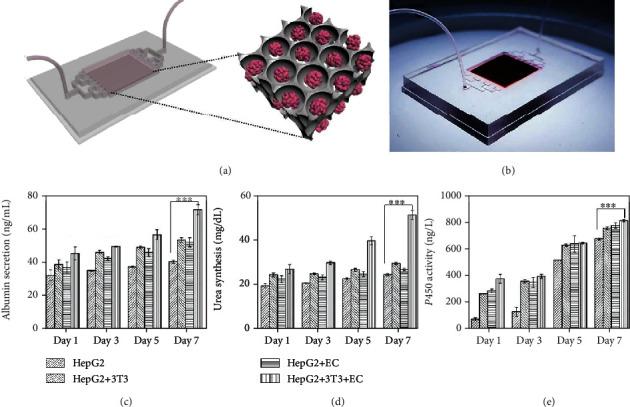
The applications of the GO hydrogel scaffolds in a liver-on-a-chip system. (a) Schematic of the construction of the liver-on-a-chip. (b) Image of the GO hydrogel scaffold-integrated liver-on-a-chip. (c–e) Albumin secretion (c), urea synthesis (d), and cytochrome P450 expression (e) of HepG2 after coculture with 3T3, ECs, and both 3T3 and ECs in the liver-on-a-chip system for 7 days; ^∗∗∗^*p* < 0.01.
