# Supplementary material for: Responsive Inverse Opal Scaffolds with Biomimetic Enrichment Capability for Cell Culture
Source: Research (Wash D C). 2019 Oct 29;2019:9783793. doi: 10.34133/2019/9783793 (PMC6946249; doi:10.34133/2019/9783793)
Supplement: Supplementary 1 — Figure S1: the real-time microscopic images of the generation of single-emulsion droplets and the relationship between the droplet diameter and the flow rate. Figure S2: the photothermally responsive behavior of the GO/NIPAM composite hydrogel. Figure S3: the fluorescent images and the MTT result of the HepG2 cells cultured on multiwell plates and the GO/NIPAM hydrogel membranes. Figure S4: the size of the pores and the interconnected porous structure before and after NIR radiation and the distribution of the cells in the pores after 1, 3, and 5 cycles. Figure S5: the images of the HepG2 spheroid formation in scaffolds on day 1, day 4, and day 7 of culture and the MTT result of the HepG2 spheroid cultured for 7 days, compared with the multiwell plate. Figure S6: the confocal images of the HepG2 spheroid in different Z-planes. Figure S7: SEM images of the cell spheroid. Figure S8: the confocal images of calcein-AM-stained ECs after coculture with 3T3 for 7 days. Figure S9: the construction of the liver-on-a-chip. [file 9783793.f1.doc]

Supplementary Materials for

**Responsive inverse opal scaffolds with biomimetic enrichment capability for cell culture**

Changmin Shao, Yuxiao Liu, Junjie Chi, Jie Wang, Ze Zhao, and Yuanjin Zhao*

Figure S1. The real-time microscopic images of the generation of single emulsion droplets and the relationship between the droplet diameter and the flow rate.

Figure S2. The photothermally responsive behavior of the GO/NIPAM composite hydrogel.

Figure S3. The fluorescent images and the MTT result of the HepG2 cells cultured on multi-well plate and the GO/NIPAM hydrogel membranes.

Figure S4. The size of pores and interconnected porous structure before and after NIR radiation and the distribution of the cells in the pores after 1, 3 and 5 cycles.

Figure S5. The images of HepG2 spheroid formation in scaffolds on day 1, day 4 and day 7 culture and the MTT result of HepG2 spheroid cultured for 7 days, compared with multi-well plate.

Figure S6. The confocal images of HepG2 spheroid in different Z-planes.

Figure S7. SEM images of cell spheroid.

Figure S8. The confocal images of calcein-AM stained ECs after co-culture with 3T3 for 7 days.

Figure S9. The construction of the liver-on-a-chip.

Movie S1. The inverse opal GO hydrogel scaffold under NIR light.


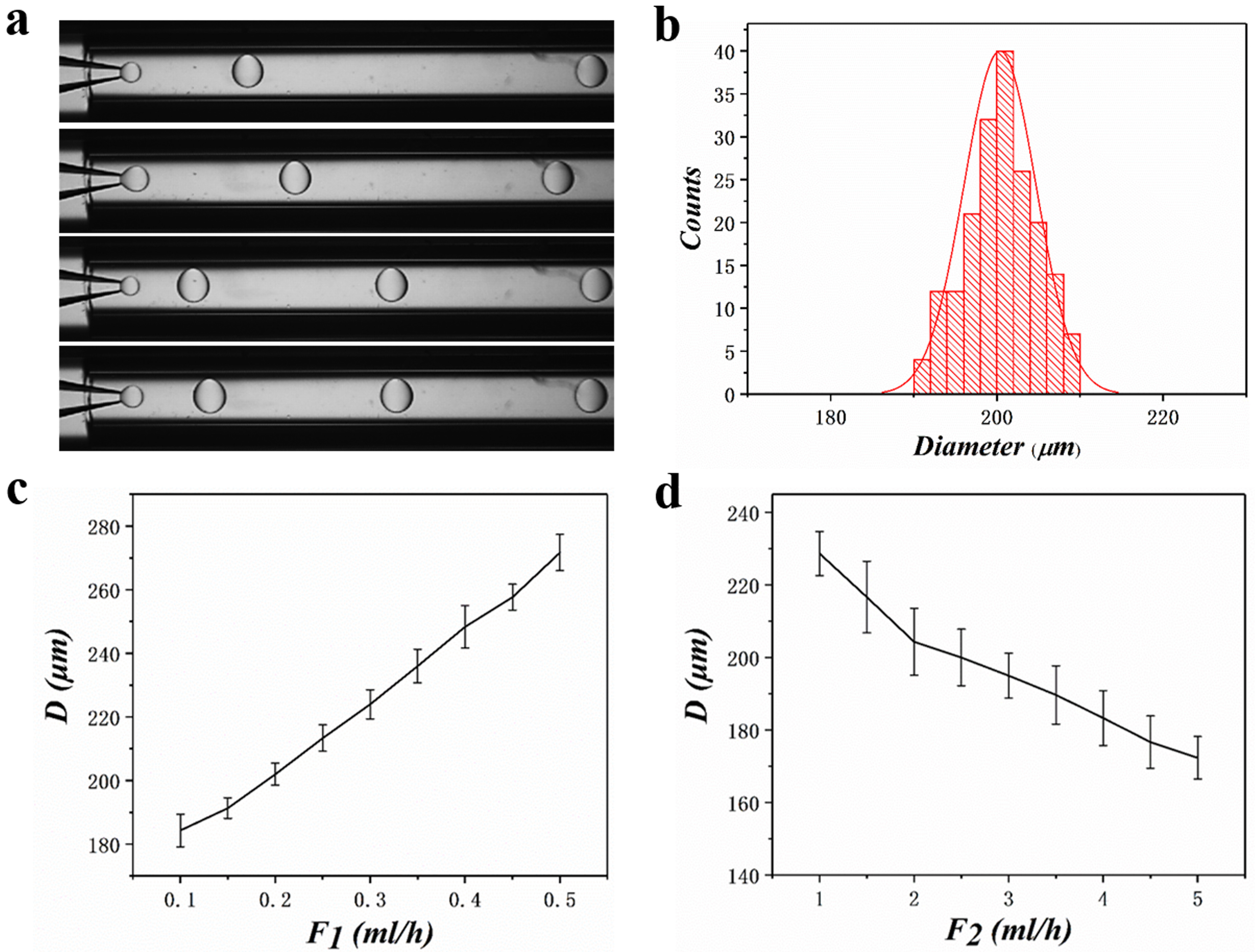


Figure S1. (a) Real-time microscopic images of the generation of single emulsion droplets. (b) The size distribution of the droplets. (c) The relationship between the droplet diameter and the inner flow rate (F1). (d) The relationship between the droplet diameter and the outer flow rate (F2).


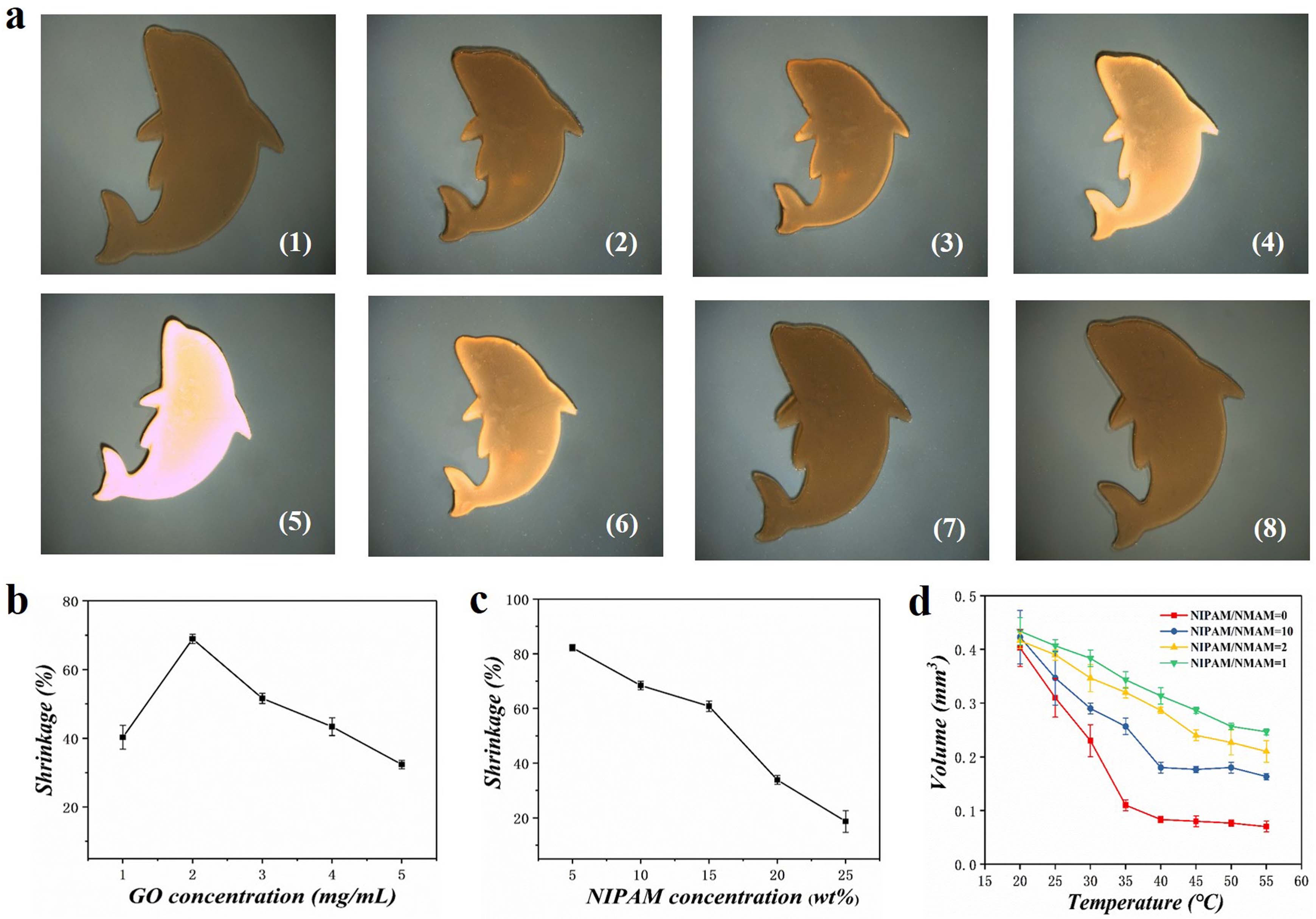


**Figure S2.** (a) The photothermally responsive behavior of the GO/NIPAM composite hydrogel, (1-5) the hydrogel gradually shrank under the NIR radiation; (6-8) the hydrogel gradually swelled and returned to the original state when the NIR radiation was switched off. (b, c) The effects of the concentrations of GO b) and NIPAM c) components on the volume shrinkage ratio of the composite hydrogel. (d) Volume size of the GO/NIPAM composite hydrogel at different temperatures. The LCST of NIPAM could be tailored by adding different concentration of NMAM into the hydrogel.


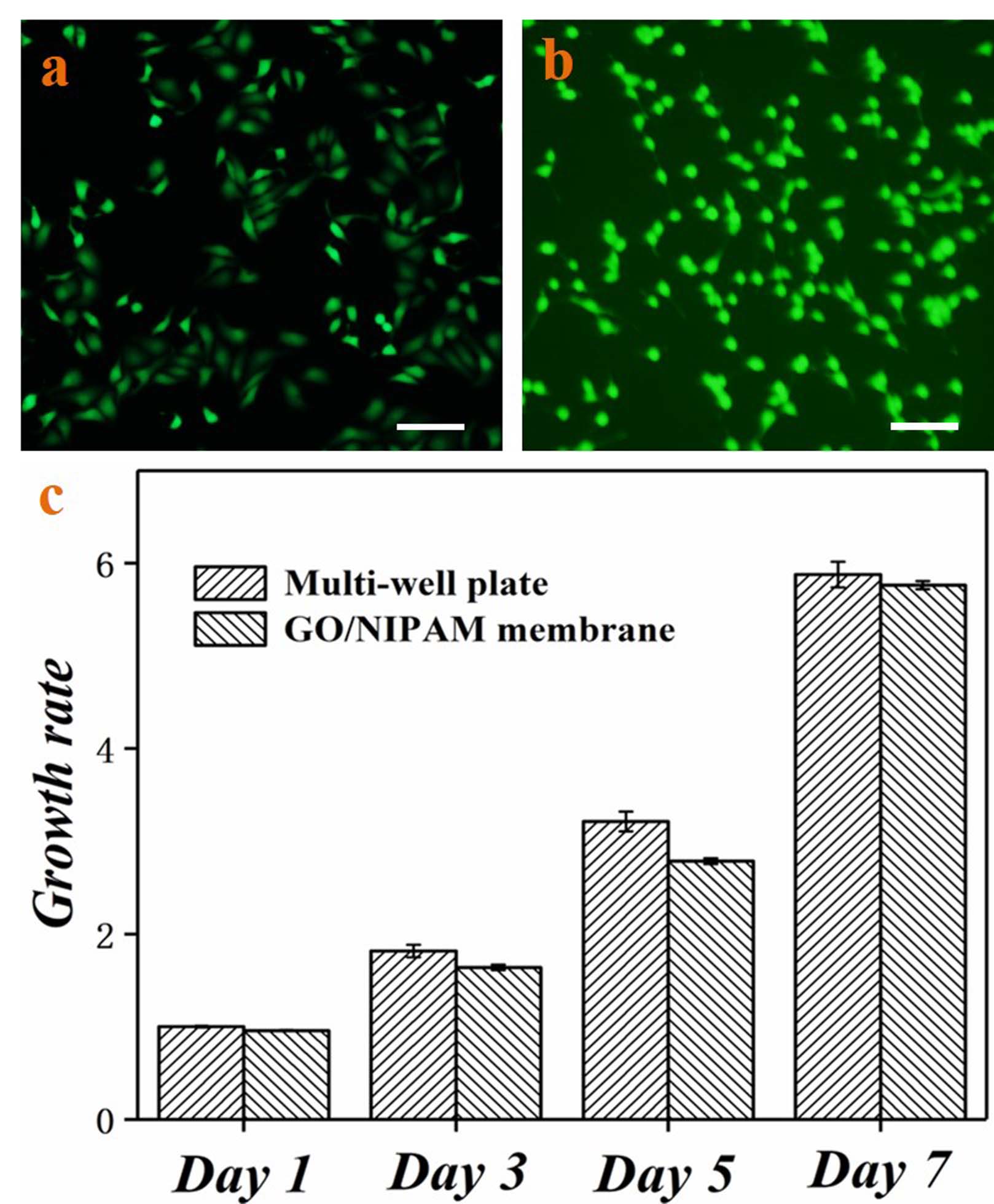


**Figure S3.** (a, b) Fluorescent images of the HepG2 cells cultured on multi-well plate a) and the GO/NIPAM hydrogel membranes b). (c) Results of the MTT assay of the HepG2 cells cultured on multi-well plate and the GO/NIPAM hydrogel membranes for 7 days. The scale bar is 50 μm.


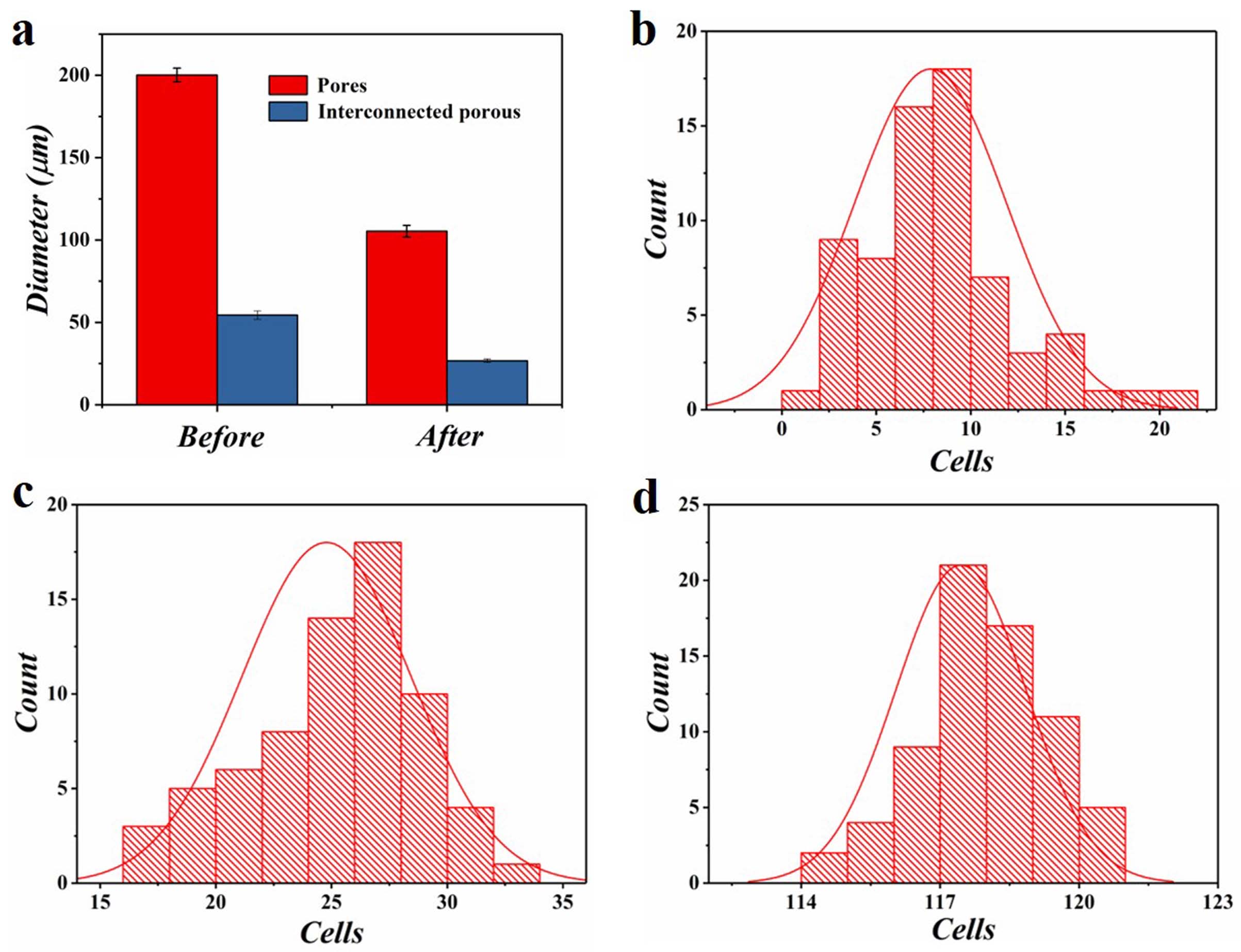


**Figure S4.** (a) The size of pores and interconnected porous structure before and after NIR radiation. (b-d) The distribution of the cells in the pores after 1 b), 3 c) and 5 d) cycles.


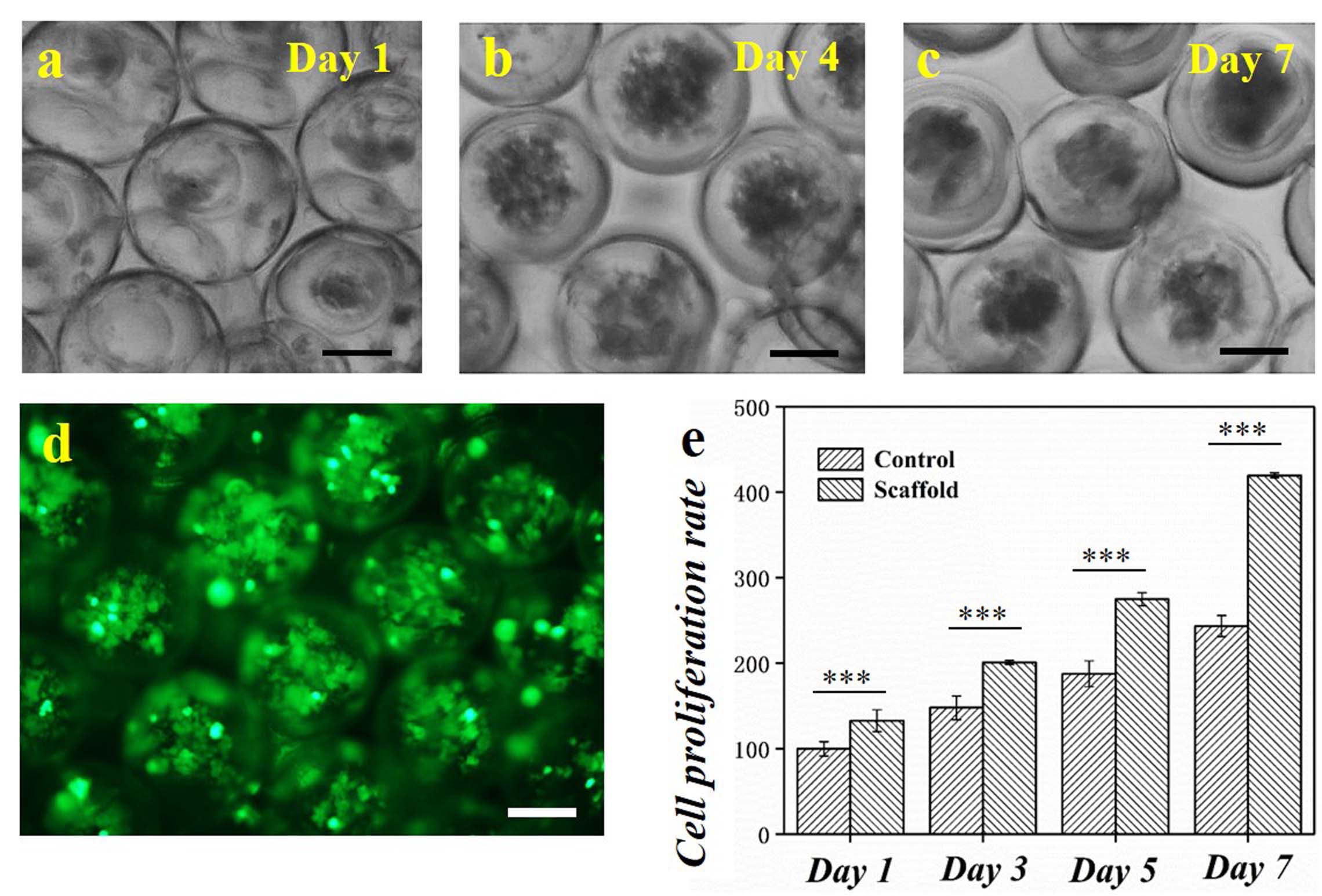


**Figure S5.** (a-c) Optical images of HepG2 spheroid formation in scaffolds on day 1, day 4 and day 7 culture. (d) the fluorescent image of the HepG2 spheroid on day 7. (e) The MTT result of HepG2 spheroid cultured for 7 days, compared with multi-well plate, ***p < 0.01. The scale bar is 100 μm.


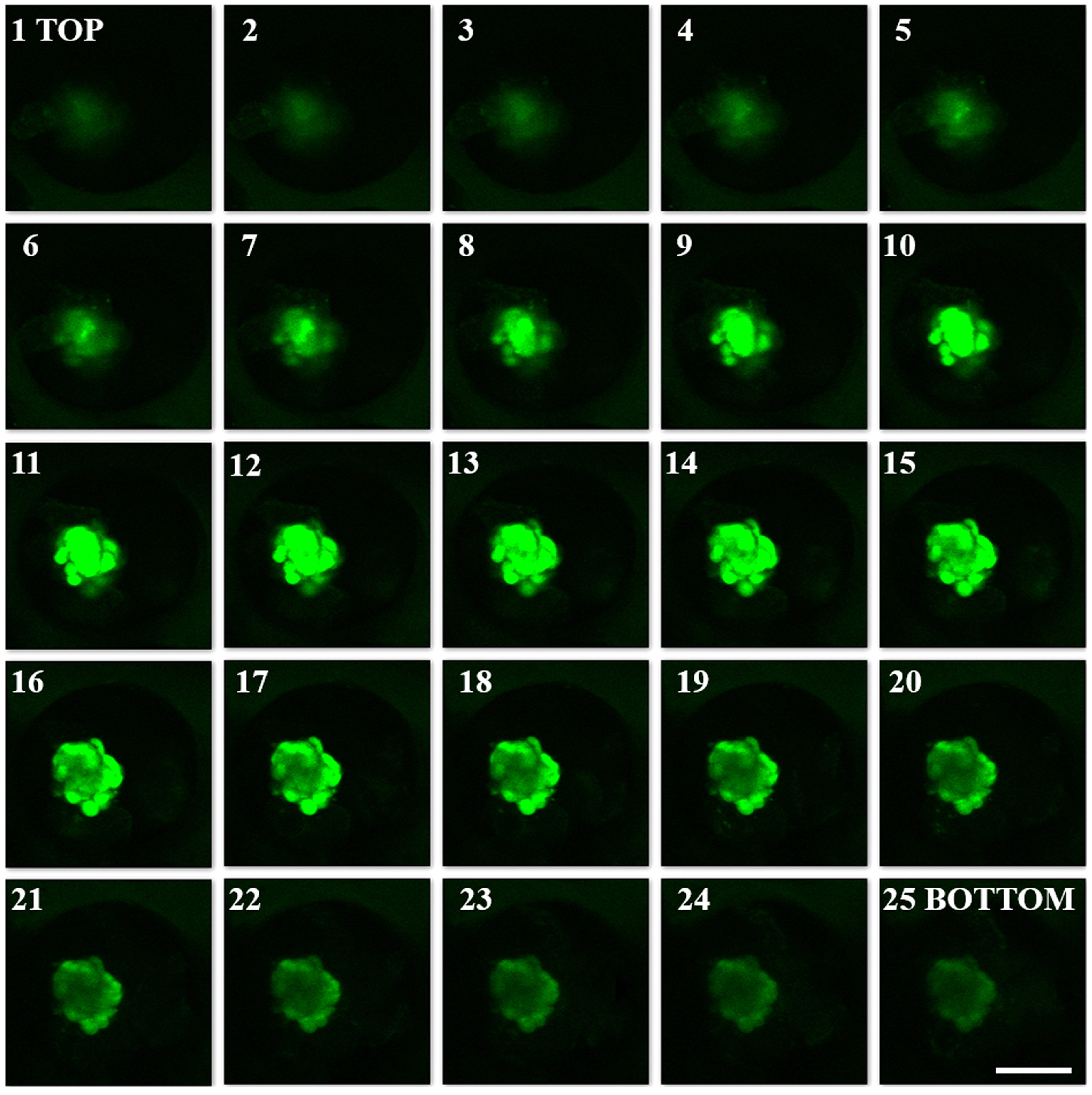


**Figure S6.** The confocal images of HepG2 spheroid in different Z-planes. The scale bar is 200 μm.


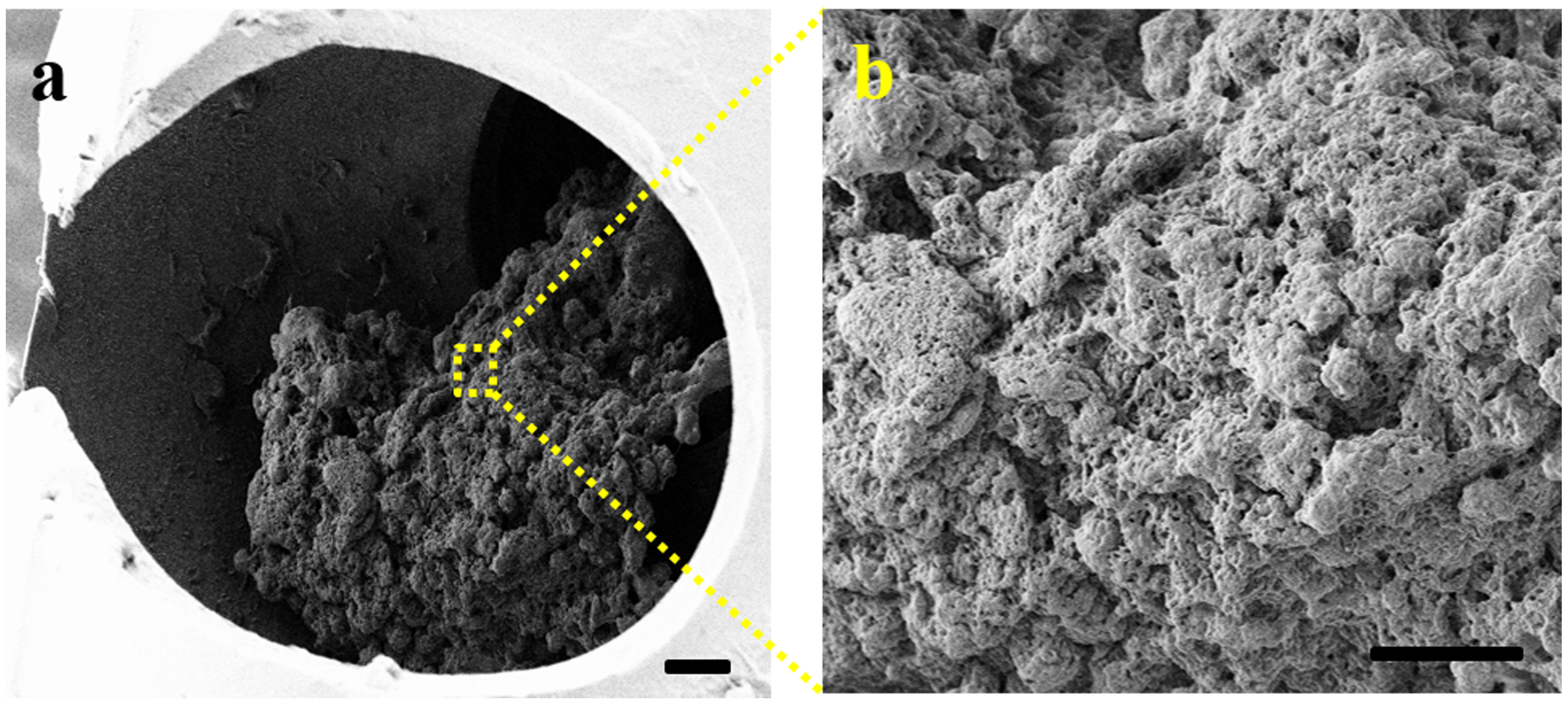


**Figure S7.** SEM images of cell spheroid. The scale bars are 20 and 2 μm in a) and b), respectively.


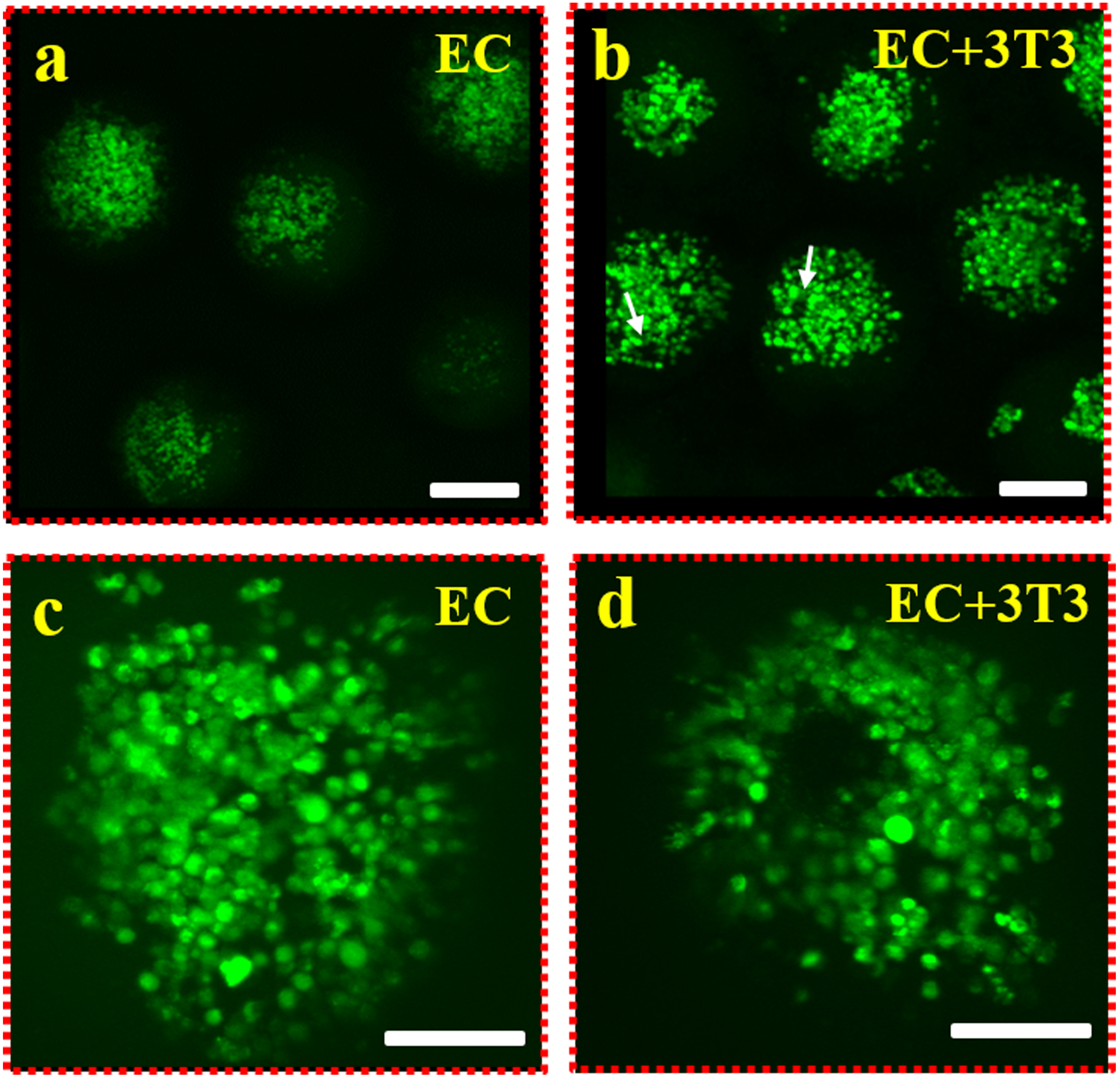


**Figure S8.** (a-d) The confocal images of calcein-AM stained ECs after co-culture with 3T3 for 7 days. White arrows indicate capillary-like structures. The scale bar is 100 μm.


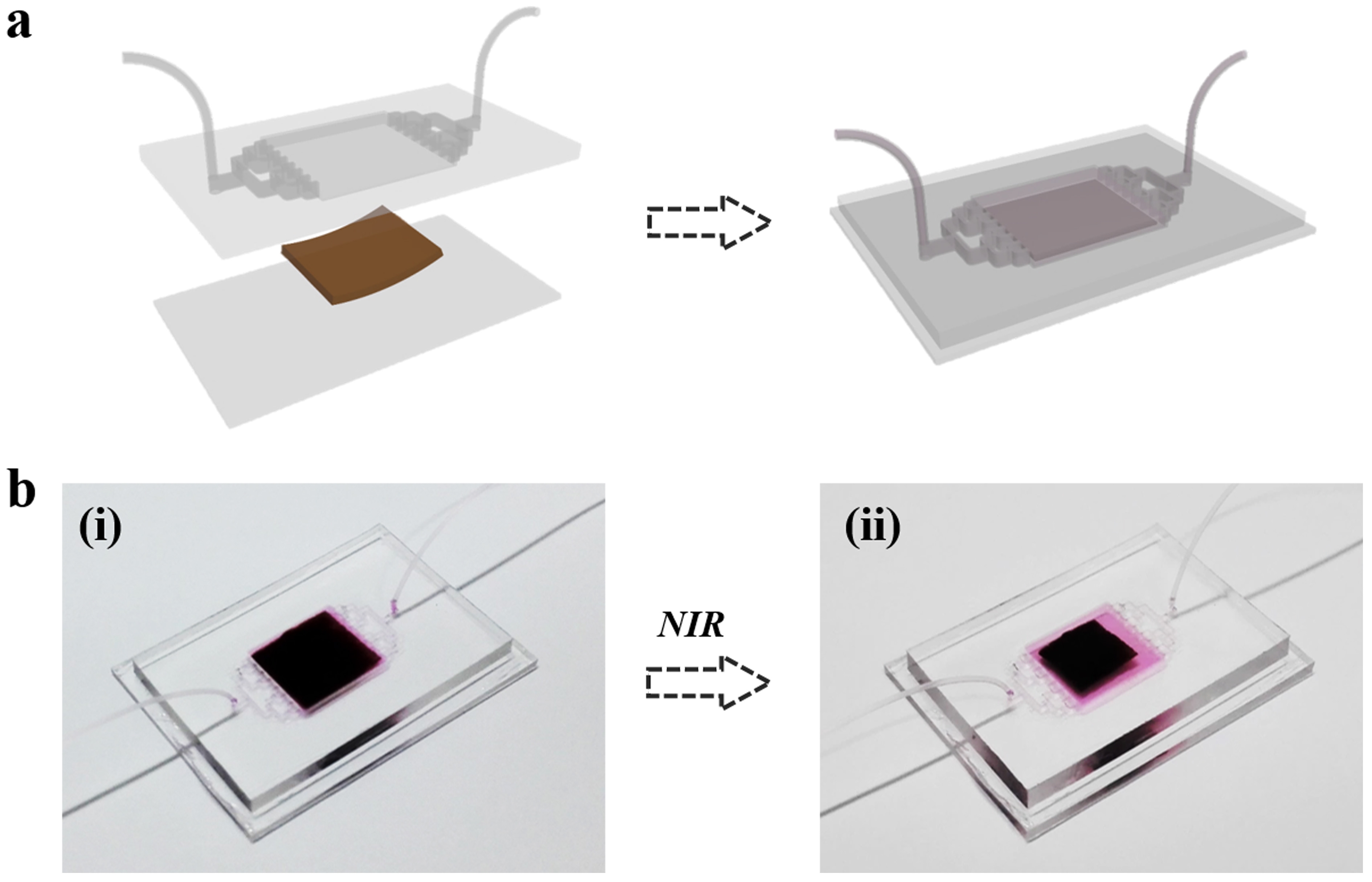


**Figure S9.** (a) Schematic of the construction of the liver-on-a-chip. (b) Image of the GO hydrogel scaffolds integrated liver-on-a-chip [normally](javascript:;) (i) and under the NIR radiation (ii).
